# Supplementary material for: From sequence to enzyme mechanism using multi-label machine learning
Source: BMC Bioinformatics. 2014 May 19;15:150. doi: 10.1186/1471-2105-15-150 (PMC4229970; doi:10.1186/1471-2105-15-150)
Supplement: Additional file 2 — Java code of ml2db. Additional file ml2db_code.tar.gz contains the Java source code to run the multi-label machine learning experiments and save the results to database. The code’s Javadoc is included. [file 1471-2105-15-150-S2.zip › additional file 2/ml2db/ml2db/doc/mulan/classifier/meta/HMC.html]

HMC


JavaScript is disabled on your browser.


- Overview
- Package
- Class
- Use
- Tree
- Deprecated
- Index
- Help

- Prev Class
- Next Class

- Frames
- No Frames

- All Classes

- Summary:
- Nested |
- Field |
- Constr |
- Method

- Detail:
- Field |
- Constr |
- Method


mulan.classifier.meta

## Class HMC

- java.lang.Object
- - mulan.classifier.MultiLabelLearnerBase
  - - mulan.classifier.meta.MultiLabelMetaLearner
    - - mulan.classifier.meta.HMC

- All Implemented Interfaces:
  :   java.io.Serializable, MultiLabelLearner, weka.core.TechnicalInformationHandler

  ---

    

  ```
  public class HMC
  extends MultiLabelMetaLearner
  ```

  Class that implements a Hierarchical Multilabel classifier (HMC). HMC classifier takes as parameter any kind of multilabel classifier and builds a hierarchy. Any node of hierarchy is a classifier and is trained separately. The root classifier is trained on all data and as getting down the hierarchy tree the data is adjusted properly to each node. Firstly, instances that do not belong to the node are removed and then attributes that are unnecessary are removed also. For more information, see

  Version:
  :   2012.07.16

  Author:
  :   George Saridis, Grigorios Tsoumakas

  See Also:
  :   Serialized Form

- - ### Constructor Summary

    Constructors

    Constructor and Description

    `HMC()`

    Default constructor

    `HMC(MultiLabelLearner baseLearner)`

    Constructs a new instance
  - ### Method Summary

    Methods

    | Modifier and Type | Method and Description |
    | --- | --- |
    | `long` | `getNoClassifierEvals()` Reurns number of classifier evaluations |
    | `long` | `getNoNodes()` Returns the number of nodes |
    | `weka.core.TechnicalInformation` | `getTechnicalInformation()` Returns an instance of a TechnicalInformation object, containing detailed information about the technical background of this class, e.g., paper reference or book this class is based on. |
    | `long` | `getTotalUsedTrainInsts()` Returns number of total instances used |
    | `java.lang.String` | `globalInfo()` Returns a string describing the multi-label learner. |

    - ### Methods inherited from class mulan.classifier.meta.MultiLabelMetaLearner

      `getBaseLearner`
    - ### Methods inherited from class mulan.classifier.MultiLabelLearnerBase

      `build, getDebug, isUpdatable, makeCopy, makePrediction, setDebug`
    - ### Methods inherited from class java.lang.Object

      `equals, getClass, hashCode, notify, notifyAll, toString, wait, wait, wait`

- - ### Constructor Detail


    - #### HMC

      ```
      public HMC()
      ```

      Default constructor


    - #### HMC

      ```
      public HMC(MultiLabelLearner baseLearner)
      ```

      Constructs a new instance

      Parameters:
      :   `baseLearner` - the multi-label learner at each node
  - ### Method Detail


    - #### getTechnicalInformation

      ```
      public weka.core.TechnicalInformation getTechnicalInformation()
      ```

      **Description copied from class: `MultiLabelLearnerBase`**

      Returns an instance of a TechnicalInformation object, containing detailed
      information about the technical background of this class, e.g., paper
      reference or book this class is based on.

      **Specified by:**
      :   `getTechnicalInformation` in interface `weka.core.TechnicalInformationHandler`

      **Specified by:**
      :   `getTechnicalInformation` in class `MultiLabelLearnerBase`

      Returns:
      :   the technical information about this class


    - #### getNoNodes

      ```
      public long getNoNodes()
      ```

      Returns the number of nodes

      Returns:
      :   number of nodes


    - #### getNoClassifierEvals

      ```
      public long getNoClassifierEvals()
      ```

      Reurns number of classifier evaluations

      Returns:
      :   number of classifier evaluations


    - #### getTotalUsedTrainInsts

      ```
      public long getTotalUsedTrainInsts()
      ```

      Returns number of total instances used

      Returns:
      :   total instances used


    - #### globalInfo

      ```
      public java.lang.String globalInfo()
      ```

      **Description copied from class: `MultiLabelLearnerBase`**

      Returns a string describing the multi-label learner.

      **Specified by:**
      :   `globalInfo` in class `MultiLabelLearnerBase`

      Returns:
      :   a description suitable for displaying in a future gui


- Overview
- Package
- Class
- Use
- Tree
- Deprecated
- Index
- Help

- Prev Class
- Next Class

- Frames
- No Frames

- All Classes

- Summary:
- Nested |
- Field |
- Constr |
- Method

- Detail:
- Field |
- Constr |
- Method
